# Syntheses and crystal structures of a nitro–anthracene–isoxazole and its oxidation product

**DOI:** 10.1107/S2056989022005710

**Published:** 2022-06-10

**Authors:** Chun Li, Matthew J. Weaver, Michael J. Campbell, Nicholas R. Natale

**Affiliations:** a Department of Chemistry, Ithaca College, 953 Danby Road, Ithaca, NY 14850, USA; b Department of Biomedical and Pharmaceutical Sciences, University of Montana, Missoula, MT 59812, USA

**Keywords:** crystal structure, isoxazole, anthracenyl isoxazole, oxidation product

## Abstract

The syntheses and structures of an unexpected by-product from an iodination reaction, namely, ethyl 5-methyl-3-(10-nitro­anthracen-9-yl)isoxazole-4-carb­oxy­l­ate, C_21_H_16_N_2_O_5_, (I), and its oxidation product, ethyl 3-(9-hy­droxy-10-oxo-9,10-di­hydro­anthracen-9-yl)-5-methyl­isoxazole-4-carboxyl­ate, C_21_H_17_NO_5_ (V) are described. Compound (I) crystallizes with two mol­ecules in the asymmetric unit in which the dihedral angles between the anthracene fused-ring systems and isoxazole ring mean planes are 88.67 (16) and 85.64 (16)°; both mol­ecules feature a disordered nitro group. In (V), which crystallizes with one mol­ecule in the asymmetric unit, the equivalent dihedral angle between the almost planar anthrone ring system (r.m.s. deviation = 0.029 Å) and the pendant isoxazole ring is 89.65 (5)°. In the crystal of (I), the mol­ecules are linked by weak C—H⋯O inter­actions into a three-dimensional network and in the extended structure of (V), inversion dimers linked by pairwise O—H⋯O hydrogen bonds generate *R*_2_^2^(14) loops.

## Abstract 2

The title compounds arose as unexpected by-products of an iodination reaction: in each case the fused-ring and isoxazole planes are almost perpendicular to each other.

## 1. Chemical context

In the course of our study of aryl-isoxazole amide (AIM) anti-tumor agents, we have a standard operating procedure to identify by-products of the synthesis (Weaver, Campbell *et al.*, 2020 ▸), and have used the mechanistic insights gained in order to optimize and improve subsequent syntheses. 
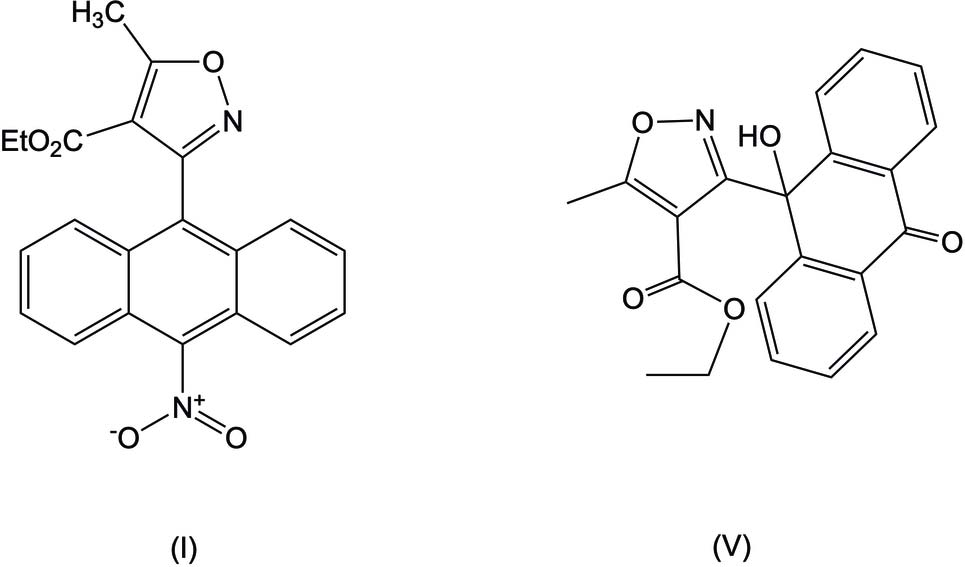


During recent structure–activity relationship studies, we encountered complications in constructing sterically hindered examples, which we desired for their calculated pharmacokinetic properties. After obtaining mediocre results with bromine as a leaving group in Suzuki couplings, we pursued a fairly routine alternative of moving to the next halogen down in the periodic table. We have encountered more complications in this study than in the previous twenty papers we have published in this area (*e.g.* Weaver, Stump *et al.*, 2020 ▸ and Weaver *et al.*, 2015 ▸), and herein report the crystal structures of two compounds observed.

**Figure 1:**
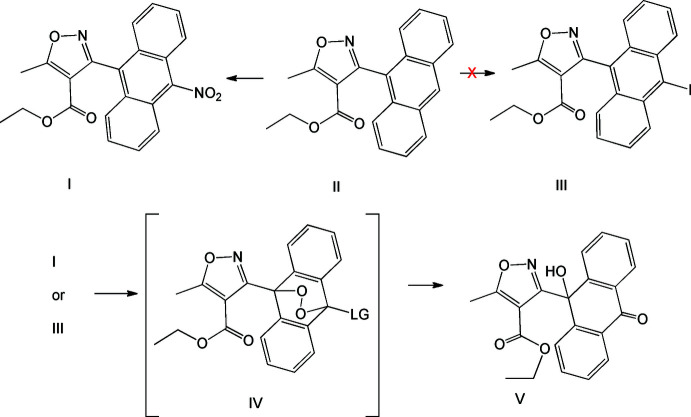
Preparation and mol­ecular structures of the title compounds.

Using conditions usually reported for iodination, the main product observed for reaction of (II) was the nitro ester (I) rather than the expected iodo product (III), which was obtained in small amounts (Fig. 1 ▸). The nitro product so obtained exhibits most of the stereoelectronic properties of previously studied analogues that we have considered to be essential for their biological activity (Han *et al.*, 2009 ▸). The nitro group is disordered and found in two distinct conformations in the unit cell. We attribute this to an extreme *peri*-effect, which substanti­ally raises the energy of the co-planar conformer.

In order to improve on the accuracy of the crystal structure of (I) we attempted numerous recrystallizations; however, what was observed was the addition of oxygen to compound (I), which we attribute to cyclo­addition of di­oxy­gen to an *endo*-peroxide (IV) (Klaper *et al.*, 2016 ▸), and ring opening with loss of a leaving group to the oxidation product anthra­quinone (V). Usually, anthracenes are oxidized *in vivo* predominantly by cytochrome P450, leading to a potentially toxic arene oxide (Silverman *et al.*, 2014 ▸). The rationale for the isoxazole series is that the C-5 isoxazole methyl group represents an opportunity for safer metabolism (Natale *et al.*, 2010 ▸). The observation in this manuscript suggests that intra­molecular di­oxy­genation, which would likely be mediated *in vivo* by mono amine oxidase (MAO), is another plausible route (Silverman, 2002 ▸). The observation of a possible *endo*-peroxide pathway in this study suggests that the metabolism of these 10-substituted anthracenyl isoxazole analogues could go through di­oxy­genation catalysed by COX (cyclo­oxygenase) and other prostaglandin synthases *in vivo* (Silverman, 2002 ▸).

## 2. Structural commentary

**Figure 2:**
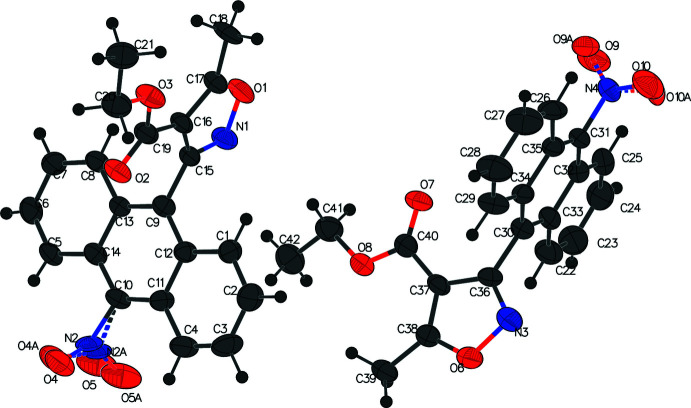
The asymmetric unit of compound (I) showing displacement ellipsoids drawn at the 50% probability level. The structure on the left is mol­ecule *A* and that on the right is mol­ecule *B*.

The first title compound (I), C_21_H_16_N_2_O_5_, crystallizes in the monoclinic *Cc* space group with two independent mol­ecules in the asymmetric unit (Fig. 2 ▸). The dihedral angle between the anthracene ring mean plane and the isoxazole ring mean plane indicate near orthogonality: 88.67 (16) and 85.64 (16)° for mol­ecules *A* (containing C1) and *B* (containing C22), respectively. Each independent anthryl ring contains a 10-nitro group with the O atoms disordered over two orientations. The isoxazole group and its attached ethyl ester moiety are virtually co-planar, with the twist angles found to be 3.1 (2)° between the C15–C17/O1/N1 and O2/C19/O3/C20 planes in mol­ecule *A*, and 4.2 (2)° between the C36–C38/O6/N3 and O7/C40/O8/C41 planes in mol­ecule *B*. The ester ethyl group is *exo-* with respect to the anthryl ring in the solid state but this conformation is not completely retained in solution as the proton NMR indicates significant anisotropy at the methyl group of the ethyl ester (δ = 0.41), which indicates at the very least a significant population of the *endo*- orientation. In addition, many of our other reported anthracenyl isoxazole esters have shown the ester ethyl group in an *endo-* orientation (Weaver, Stump *et al.*, 2020 ▸; Weaver *et al.*, 2015 ▸; Li *et al.*, 2013 ▸; Li *et al.*, 2006 ▸; Han *et al.*, 2003 ▸; Mosher *et al.*, 1996 ▸).

**Figure 3:**
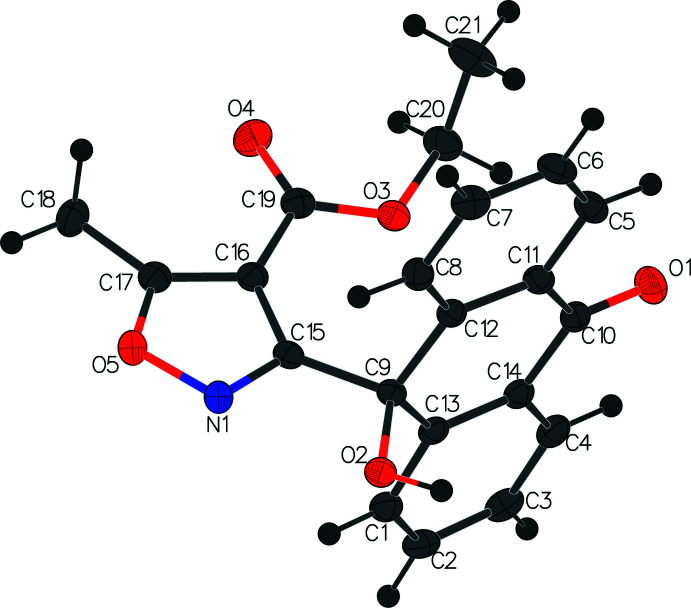
The asymmetric unit of compound (V) with displacement ellipsoids drawn at the 50% probability level.

The second title compound (V), C_21_H_17_NO_5_, crystallizes in the monoclinic *P*2_1_/*c* space group with one independent mol­ecule in the asymmetric unit (Fig. 3 ▸). The anthrone ring system is virtually planar with an r.m.s. deviation of 0.029 Å. Like the other anthracenyl isoxazole structures we have reported (*vide supra*), the isoxazole ring is orthogonal to the anthracene ring, with a dihedral angle of 89.65 (5)°. The ester ethyl group is in *endo-* orientation and the C19—O3—C20—C21 grouping is twisted [torsion angle = 86.7 (2)°].

## 3. Supra­molecular features

**Figure 4:**
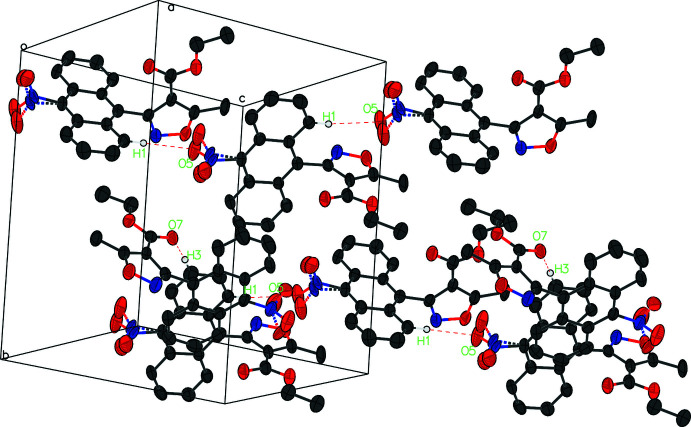
The partial packing of compound (I). For clarity, only hydrogen bonds C1—H1⋯O5^i^ and C3—H3⋯O7^ii^ are shown as dashed lines, and H atoms not involved in these hydrogen bonds are removed.

In compound (I), weak C—H⋯O hydrogen bonds between adjacent *A* mol­ecules (C7—H7⋯O4 and C1—H1⋯O5) form a column running perpendicular to the [101] direction. Mol­ecule *B* lies between the columns and its O7 atom accepts a hydrogen bond from H3 of mol­ecule *A* (Table 1 ▸, Fig. 4 ▸). There is an aromatic π–π stacking inter­action with a centroid–centroid separation of 3.537 (5) Å between the planes of the C22–C25/C32/C33 and C1–C4/C11/C12 rings. A σ–π inter­action is observed at a distance of 3.774 Å from atom C42 to the plane centroid.

**Figure 5:**
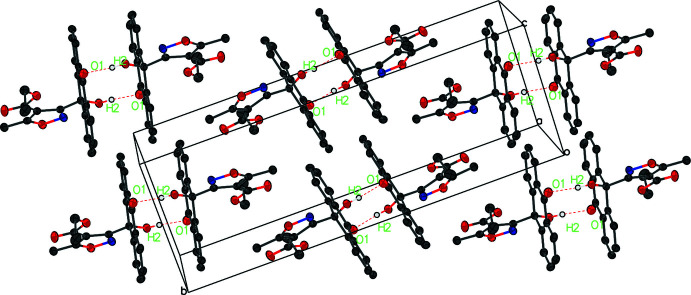
The packing of compound (V). Inversion dimers linked by pairwise O2—H2⋯O1 hydrogen bonds are shown in dashed lines.

In the crystal of compound (V), inversion dimers linked by pairwise O2—H2⋯O1 hydrogen bonds occur (Table 2 ▸, Fig. 5 ▸). A short contact distance between the isoxazole ring of one mol­ecule (ring mean plane C15–C17/O5N1) and the carbonyl oxygen (O4) of another mol­ecule [3.1486 (16) Å] may contribute to the head-to-head, tail-to-tail arrangement in the crystal structure, also shown in Fig. 8 ▸ *b*.

## 4. Hirshfeld surface analysis

**Figure 6:**
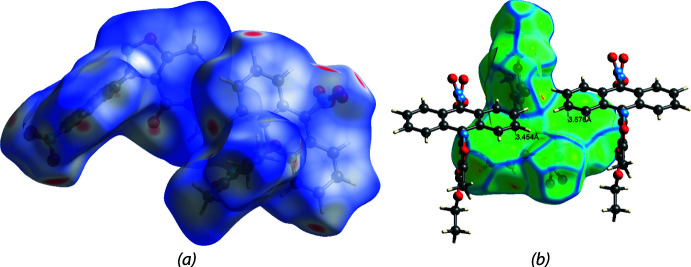
(*a*) The Hirshfeld surface of (I) mapped over *d*_norm_. Short and long contacts are indicated as red and blue spots, respectively. Contacts with distances approximately equal to the sum of the van der Waals radii are colored white. (*b*) Weak π–π inter­actions are shown as green dashed lines on a surface mapped over curvedness. The π–π stacking is indicated by the green flat regions surrounded by dark blue edges.

**Figure 7:**
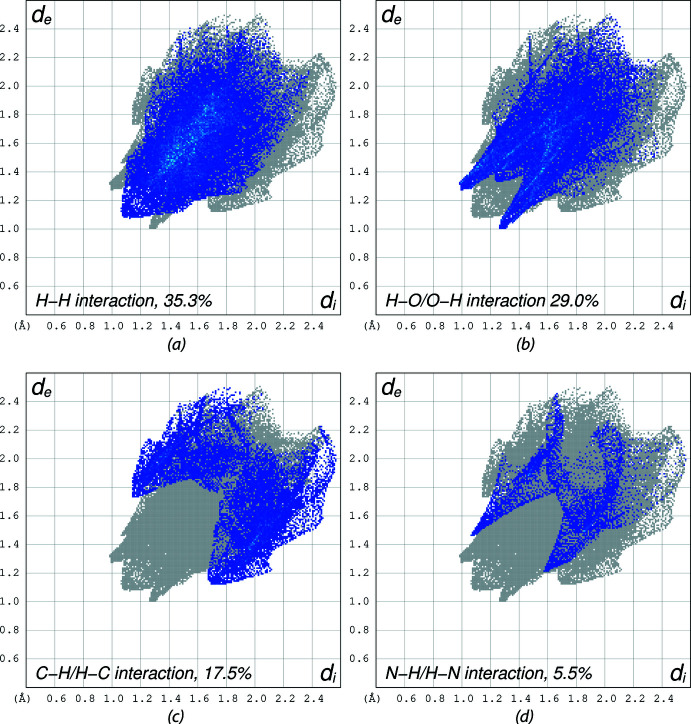
The two-dimensional fingerprint plots for (I) delineated into (*a*) H⋯H contacts, (*b*) O⋯H/H⋯O contacts, (*c*) C⋯H/H⋯C contacts, and (*d*) N⋯H/H⋯N contacts. Other contact contributions less than 5% are omitted.

Hirshfeld surface analysis (Spackman & Jayatilaka, 2009 ▸) was performed, and the associated two-dimensional fingerprint plots (McKinnon *et al.*, 2007 ▸) were generated to qu­antify the inter­molecular inter­actions using Crystal Explorer 21.5 (Spackman *et al.*, 2021 ▸). The Hirshfeld surface of (I) is mapped over *d*_norm_ in a fixed color scale of −0.31 (red) to 1.26 (blue) arbitrary units (Fig. 6 ▸). The delineated two-dimensional fingerprint plots shown in Fig. 7 ▸ indicate that two main contributions to the overall Hirshfeld surface area arise from H⋯H contacts (35.3%) and O⋯H/H⋯O contacts (29.0%) with C⋯H/H⋯C inter­actions contributing 17.5% of the Hirshfeld surface.

**Figure 8:**
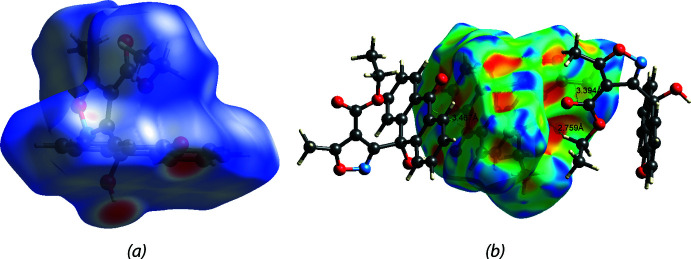
(*a*) The Hirshfeld surface of (V) mapped over *d*_norm_. Short and long contacts are indicated as red and blue spots, respectively. Contacts with distances approximately equal to the sum of the van der Waals radii are colored white. Hydroxyl and carbonyl groups on the anthrone ring contributed major short contacts. (*b*) π–π inter­actions (anthrone to anthrone and carbonyl to isoxazole ring) and σ–π inter­action (C—H bond to carbon­yl) are shown as orange–red spots with green dashed lines in the shape-index map.

**Figure 9:**
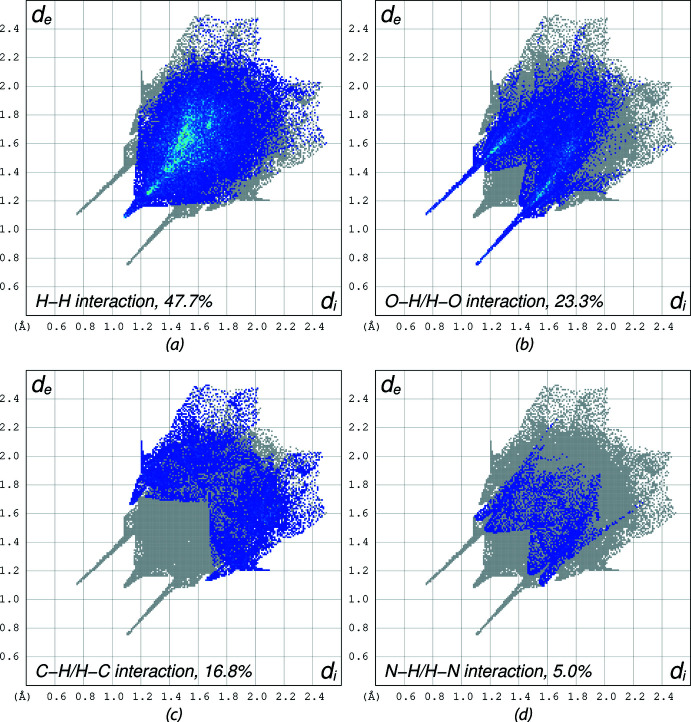
The two-dimensional fingerprint plots for (V) delineated into (*a*) H⋯H contacts, (*b*) O⋯H/H⋯O contacts, (*c*) C⋯H/H⋯C contacts, and (*d*) N⋯H/H⋯N contacts. Other contact contributions less than 5% are omitted.

The Hirshfeld surface of compound V is mapped over *d*_norm_ in a fixed color scale of −0.58 (red) to 1.31 (blue) arbitrary units (Fig. 8 ▸ *a*), showing two short contacts from O⋯H hydrogen bonds in red spots. The delineated two-dimensional fingerprint plots (Fig. 9 ▸) indicate that H⋯H contacts contribute 47.7% of the Hirshfeld surface. Aromatic π–π stacking is also identifiable from the Hirshfeld surface mapped over the shape-index property (Fig. 8 ▸ *b*).

## 5. Database survey

A search for the 9-nitro­anthracenyl moiety in the Cambridge Structural Database (CSD version 5.43, November 2021 update; Groom *et al.*, 2016 ▸) resulted in 14 hits, of which two crystal structures of 9-nitro­anthracene itself were reported, namely refcodes NTRANT (Trotter, 1959 ▸) and NTRANT01 (Glagovich *et al.*, 2004 ▸). The reported angles between the NO_2_ plane and the anthracene plane are 84.78 and 69.40°, respectively, which agree with our observation of the disordered NO_2_ group in (I).

A search in the same database for the 10-hy­droxy anthrone fragment resulted in 59 hits, of which 10 structures had an aromatic ring at the 10-position, namely refcodes COBWEY (Barker *et al.*, 2019 ▸), DULVUB (Skrzat & Roszak, 1986 ▸), ELULII (Stepovik *et al.*, 2015 ▸), EVETIL (Mao *et al.*, 2021 ▸), JAYPAA (Roszak *et al.*, 1990 ▸), MOTJIQ (Chen *et al.*, 2015 ▸), MOTKEN (Chen *et al.*, 2015 ▸), QAJPUQ (Forensi *et al.*, 2020 ▸), SAMNEC (Hoffend *et al.*, 2013 ▸) and WOKYIH (Pullella *et al.*, 2019 ▸). The anthrone unit in these 10 structures are either essentially planar or in a shallow boat conformation. The aromatic rings at the 10-position in these compounds are all at a vertical orientation relative to the anthrone ring. It may be noted that an anthrone isoxazole ester we reported in 2014, refcode TIYZEI, also shares similar structural features (Duncan *et al.*, 2014 ▸).

## 6. Synthesis and crystallization

Iodination of aromatic hydro­carbons with mol­ecular iodine has been accomplished by several methods, typically using an oxidizing agent to generate the iodo­nium cation electrophile. Among the conditions we surveyed, fuming nitric acid in particular (Bansal *et al.*, 1987 ▸) with the anthracene isoxazole (II), appears to consistently produce the nitrated anthryl (I) rather than the desired iodo product (III). The anthryl isoxazole ester (II) was prepared as previously described (Mosher *et al.*, 1996 ▸), and recrystallized before use. The ester (II) (67 mg, 0.19 mmol) was dissolved in acetic acid (1 ml), and iodine (24.1 mg) was added. To this solution was added concentrated sulfuric acid (1 ml) and sodium nitrite (13.1 mg, 0.19 mmol). The resulting solution was warmed to reflux for 30 minutes, after which it was poured over ice (3 g) and the precipitate collected by filtration. Silica gel chromatography using ethyl acetate in hexane provided the product, which was recrystallized from solutions in methyl­ene chloride, ethyl acetate and hexane by slow evaporation, whereby the product was obtained as dull dark-yellow prisms (15 mg, 21%). ^1^H NMR: (CDCl_3_) δppm 7.95 (*d*, 2H, *J* = 8Hz); 7.69 (*m*, 4H); 7.6 (*m*, 2H); 3.735 (*q*, 2H, *J* = 4Hz); 2.94 (*s*, 3H); 0.41 (*t*, 3H, *J* = 4Hz). ^13^C NMR: (CDCl_3_) δppm 176.66, 161.03, 159.45, 145.97, 133.59, 130.34, 128.68, 127.11, 125.67, 121.81, 121.57, 111.45, 60.41, 13.47, 12.94. HPLC–MS: calculated for [C_21_H_16_N_2_O_5_+H]^+^ 377.1137, observed *m*/*z* 377 ([*M* + 1]^+^, 100% rel. intensity).

During the re-crystallization of compound (I), different solvent combinations of hexane, methanol, di­chloro­methane, and ethyl acetate were used. Instead of better crystals of compound (I), compound (V) was formed as translucent light-yellow prisms from the slow evaporation of the solvent mixture composed of hexane and methanol at room temperature over a period of two months. ^1^H NMR: (CDCl_3_) δppm 8.29 (*dd*, 2H, *J* = 1.37 and 7.79 Hz); 7.67 (*d*, 2H, *J* = 7.79 Hz); 7.60 (*ddd*, 2H, *J* = 1.37, 7.33, and 7.79 Hz); 7.50 (*ddd*, 2H, *J* = 1.37, 7.33, and 7.79 Hz); 4.06 (*q*, 2H, *J* = 6.87 Hz); 2.60 (*s*, 3H); 1.06 (*t*, 3H, *J* = 6.87 Hz). ^13^C NMR: (CDCl_3_) δppm 183.86, 177.58, 167.05, 162.74, 143.92, 133.68, 130.96, 128.86, 127.29, 126.72, 71.26, 61.71, 14.16, 13.96.

## 7. Refinement

Crystal data, data collection and structure refinement details are summarized in Table 3 ▸. In compound (I), the nitro group is disordered in each of the two independent mol­ecules in the asymmetric unit. The occupancies of each disordered part were refined, converging to 0.572 (13) and 0.428 (13) for mol­ecule *A*, and 0.64 (3) and 0.36 (3) for mol­ecule *B*. EADP constraints were applied (Sheldrick, 2015 ▸) to each nitro group. The C-bound hydrogen atoms on both compounds were fixed geometrically and treated as riding with C—H = 0.95–0.98 Å and refined with *U*_iso_(H) = 1.2*U*_eq_(CH, CH_2_) or 1.5*U*_eq_(CH_3_). The O-bound H atom in (V) was found in a difference-Fourier map and refined freely. Four reflections ( 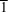 10, 110, 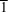 11 and 11 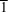 ) in compound (I) and four reflections (100, 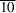 4 5, 110 and 011) in compound (V) affected by the beam stop were omitted from the final cycles of refinement because of poor agreement between the observed and calculated intensities. The absolute structure of (I) was indetermin­ate in the present refinement.

## Supplementary Material

Crystal structure: contains datablock(s) I, V, global. DOI: 10.1107/S2056989022005710/hb8020sup1.cif

Structure factors: contains datablock(s) I. DOI: 10.1107/S2056989022005710/hb8020Isup2.hkl

Structure factors: contains datablock(s) V. DOI: 10.1107/S2056989022005710/hb8020Vsup3.hkl

Supporting information file. DOI: 10.1107/S2056989022005710/hb8020Isup4.cml

Supporting information file. DOI: 10.1107/S2056989022005710/hb8020Vsup5.cml

CCDC references: 2175007, 2175006

Additional supporting information:  crystallographic information; 3D view; checkCIF report

## Figures and Tables

**Table 1:** Hydrogen-bond geometry (Å, °) for (I)

*D*—H⋯*A*	*D*—H	H⋯*A*	*D*⋯*A*	*D*—H⋯*A*
C1—H1⋯O5^i^	0.95	2.46	3.366 (12)	159
C3—H3⋯O7^ii^	0.95	2.44	3.339 (6)	158
C7—H7⋯O4^iii^	0.95	2.40	3.24 (4)	147
C7—H7⋯O4*A* ^iii^	0.95	2.46	3.34 (6)	154

Symmetry codes: (i) 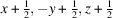 ; (ii) 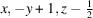 ; (iii) 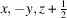 .

**Table 2:** Hydrogen-bond geometry (Å, °) for (V)

*D*—H⋯*A*	*D*—H	H⋯*A*	*D*⋯*A*	*D*—H⋯*A*
O2—H2⋯O1^i^	0.91 (3)	1.93 (3)	2.8359 (19)	176 (2)

Symmetry code: (i) 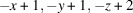 .

**Table 3:** Experimental details

	(I)	(V)
Crystal data		
Chemical formula	C_21_H_16_N_2_O_5_	C_21_H_17_NO_5_
*M*_r_	376.36	363.36
Crystal system, space group	Monoclinic, *C* *c*	Monoclinic, *P*2_1_/*c*
Temperature (K)	100	100
*a*, *b*, *c* (Å)	16.4968 (10), 14.8697 (9), 16.1836 (9)	8.2862 (4), 23.5895 (11), 8.6219 (4)
β (°)	114.879 (3)	97.728 (2)
*V* (Å^3^)	3601.5 (4)	1669.99 (14)
*Z*	8	4
Radiation type	Mo *K*α	Mo *K*α
μ (mm^−1^)	0.10	0.10
Crystal size (mm)	0.29 × 0.24 × 0.22	0.28 × 0.20 × 0.19

Data collection		
Diffractometer	Bruker SMART Breeze CCD	Bruker SMART Breeze CCD
Absorption correction	–	Numerical (*SADABS*; Krause *et al.*, 2015 ▸)
*T*_min_, *T*_max_	–	0.945, 1.000
No. of measured, independent and observed [*I* > 2σ(*I*)] reflections	45790, 7615, 5596	44252, 4112, 3252
*R*_int_	0.054	0.051
(sin θ/λ)_max_ (Å^−1^)	0.633	0.668

Refinement		
*R*[*F* ^2^ > 2σ(*F* ^2^)], *wR*(*F* ^2^), *S*	0.059, 0.158, 1.02	0.051, 0.114, 1.13
No. of reflections	7615	4112
No. of parameters	546	250
No. of restraints	2	0
H-atom treatment	H atoms treated by a mixture of independent and constrained refinement	H atoms treated by a mixture of independent and constrained refinement
Δρ_max_, Δρ_min_ (e Å^−3^)	0.55, −0.19	0.37, −0.21
Absolute structure	Flack *x* determined using 2257 quotients [(*I* ^+^)−(*I* ^−^)]/[(*I* ^+^)+(*I* ^−^)] (Parsons *et al.*, 2013 ▸)	–
Absolute structure parameter	0.5 (4)	–

Computer programs: *APEX2* (Bruker, 2012 ▸), *SAINT* (Bruker, 2018 ▸), *SHELXS* (Sheldrick, 2008 ▸), *SHELXL2018/1* (Sheldrick, 2015 ▸) and *OLEX2* (Dolomanov *et al.*, 2009 ▸).

## supplementary crystallographic information

### Ethyl 5-methyl-3-(10-nitroanthracen-9-yl)isoxazole-4-carboxylate (I) Crystal data

**Table:**

C_21_H_16_N_2_O_5_	*F*(000) = 1568
*M**_r_* = 376.36	*D*_x_ = 1.388 Mg m^−^^3^
Monoclinic, *C**c*	Mo *K*α radiation, λ = 0.71073 Å
*a* = 16.4968 (10) Å	Cell parameters from 7597 reflections
*b* = 14.8697 (9) Å	θ = 2.7–21.0°
*c* = 16.1836 (9) Å	µ = 0.10 mm^−^^1^
β = 114.879 (3)°	*T* = 100 K
*V* = 3601.5 (4) Å^3^	Prism, yellow
*Z* = 8	0.29 × 0.24 × 0.22 mm

### Ethyl 5-methyl-3-(10-nitroanthracen-9-yl)isoxazole-4-carboxylate (I) Data collection

**Table:**

Bruker SMART Breeze CCD diffractometer	*R*_int_ = 0.054
Radiation source: 2 kW sealed X-ray tube	θ_max_ = 26.7°, θ_min_ = 2.7°
φ and ω scans	*h* = −20→20
45790 measured reflections	*k* = −18→18
7615 independent reflections	*l* = −20→20
5596 reflections with *I* > 2σ(*I*)	

### Ethyl 5-methyl-3-(10-nitroanthracen-9-yl)isoxazole-4-carboxylate (I) Refinement

**Table:**

Refinement on *F*^2^	Hydrogen site location: mixed
Least-squares matrix: full	H atoms treated by a mixture of independent and constrained refinement
*R*[*F*^2^ > 2σ(*F*^2^)] = 0.059	*w* = 1/[σ^2^(*F*_o_^2^) + (0.0864*P*)^2^ + 2.331*P*] where *P* = (*F*_o_^2^ + 2*F*_c_^2^)/3
*wR*(*F*^2^) = 0.158	(Δ/σ)_max_ < 0.001
*S* = 1.02	Δρ_max_ = 0.55 e Å^−^^3^
7615 reflections	Δρ_min_ = −0.19 e Å^−^^3^
546 parameters	Absolute structure: Flack *x* determined using 2257 quotients [(*I*^+^)-(*I*^-^)]/[(*I*^+^)+(*I*^-^)] (Parsons *et al.*, 2013)
2 restraints	Absolute structure parameter: 0.5 (4)
Primary atom site location: structure-invariant direct methods	

### Ethyl 5-methyl-3-(10-nitroanthracen-9-yl)isoxazole-4-carboxylate (I) Special details

**Table:**

Geometry. All esds (except the esd in the dihedral angle between two l.s. planes) are estimated using the full covariance matrix. The cell esds are taken into account individually in the estimation of esds in distances, angles and torsion angles; correlations between esds in cell parameters are only used when they are defined by crystal symmetry. An approximate (isotropic) treatment of cell esds is used for estimating esds involving l.s. planes.

### Ethyl 5-methyl-3-(10-nitroanthracen-9-yl)isoxazole-4-carboxylate (I) Fractional atomic coordinates and isotropic or equivalent isotropic displacement parameters (Å^2^)

**Table:**

	*x*	*y*	*z*	*U*_iso_*/*U*_eq_	Occ. (<1)
O1	0.3319 (3)	0.2201 (3)	0.5466 (2)	0.0487 (9)	
O2	0.3808 (2)	0.0465 (3)	0.3397 (2)	0.0459 (9)	
O3	0.4880 (2)	0.0385 (3)	0.4815 (2)	0.0471 (9)	
N1	0.2636 (3)	0.2332 (3)	0.4569 (3)	0.0440 (10)	
C1	0.3269 (3)	0.2892 (3)	0.2833 (4)	0.0392 (11)	
H1	0.363669	0.290494	0.346904	0.047*	
C2	0.3469 (4)	0.3427 (4)	0.2259 (4)	0.0519 (14)	
H2	0.397851	0.380760	0.250073	0.062*	
C3	0.2934 (4)	0.3424 (4)	0.1320 (4)	0.0548 (15)	
H3	0.308016	0.380624	0.093210	0.066*	
C4	0.2203 (4)	0.2876 (4)	0.0955 (4)	0.0471 (13)	
H4	0.184644	0.288156	0.031651	0.056*	
C5	0.0334 (3)	0.0476 (3)	0.1397 (3)	0.0375 (11)	
H5	0.001 (4)	0.040 (3)	0.083 (4)	0.045 (15)*	
C6	0.0166 (4)	−0.0086 (4)	0.1956 (4)	0.0447 (13)	
H6	−0.030 (4)	−0.042 (4)	0.171 (4)	0.042 (15)*	
C7	0.0677 (3)	−0.0057 (4)	0.2910 (3)	0.0433 (12)	
H7	0.054540	−0.045527	0.329570	0.052*	
C8	0.1361 (3)	0.0548 (3)	0.3273 (3)	0.0363 (11)	
H8	0.170089	0.056581	0.391448	0.044*	
C9	0.2294 (3)	0.1746 (3)	0.3054 (3)	0.0335 (10)	
C10	0.1272 (3)	0.1697 (3)	0.1202 (3)	0.0359 (11)	
C11	0.1972 (3)	0.2301 (3)	0.1519 (3)	0.0354 (11)	
C12	0.2511 (3)	0.2315 (3)	0.2482 (3)	0.0326 (10)	
C13	0.1573 (3)	0.1148 (3)	0.2714 (3)	0.0295 (10)	
C14	0.1042 (3)	0.1105 (3)	0.1749 (3)	0.0325 (10)	
C15	0.2857 (3)	0.1785 (3)	0.4056 (3)	0.0330 (10)	
C16	0.3647 (3)	0.1298 (3)	0.4562 (3)	0.0353 (10)	
C17	0.3904 (3)	0.1590 (4)	0.5441 (3)	0.0404 (12)	
C18	0.4659 (4)	0.1363 (4)	0.6323 (3)	0.0537 (15)	
H18A	0.516186	0.176866	0.642774	0.081*	
H18B	0.484587	0.074058	0.630503	0.081*	
H18C	0.446894	0.143120	0.681695	0.081*	
C19	0.4107 (3)	0.0671 (4)	0.4186 (3)	0.0402 (12)	
C20	0.5375 (4)	−0.0214 (4)	0.4483 (4)	0.0517 (14)	
H20A	0.556563	0.011073	0.406087	0.062*	
H20B	0.499576	−0.072871	0.415356	0.062*	
C21	0.6175 (4)	−0.0541 (4)	0.5297 (4)	0.0585 (15)	
H21A	0.651142	−0.096711	0.509947	0.088*	
H21B	0.597864	−0.083994	0.572036	0.088*	
H21C	0.655804	−0.002855	0.560146	0.088*	
O4	0.1027 (18)	0.111 (3)	−0.022 (3)	0.072 (6)	0.572 (13)
O5	−0.0003 (7)	0.1959 (10)	−0.0102 (7)	0.068 (3)	0.572 (13)
N2	0.076 (3)	0.156 (2)	0.025 (3)	0.039 (4)	0.572 (13)
O4A	0.073 (3)	0.105 (4)	−0.022 (4)	0.072 (6)	0.428 (13)
O5A	0.0259 (10)	0.2395 (14)	−0.0113 (10)	0.068 (3)	0.428 (13)
N2A	0.066 (4)	0.178 (3)	0.017 (4)	0.039 (4)	0.428 (13)
O6	0.4350 (3)	0.7239 (3)	0.2788 (2)	0.0553 (11)	
O7	0.3756 (2)	0.5736 (2)	0.4917 (2)	0.0424 (8)	
O8	0.2920 (2)	0.5316 (2)	0.3474 (2)	0.0422 (8)	
N3	0.4850 (3)	0.7577 (3)	0.3678 (3)	0.0561 (13)	
N4	0.6061 (3)	0.7796 (3)	0.8043 (3)	0.0495 (12)	
C22	0.3713 (4)	0.8310 (4)	0.4975 (4)	0.0506 (14)	
H22	0.347573	0.821907	0.433552	0.061*	
C23	0.3270 (4)	0.8841 (4)	0.5326 (4)	0.0600 (16)	
H23	0.272392	0.911450	0.492941	0.072*	
C24	0.3606 (4)	0.8994 (4)	0.6269 (4)	0.0568 (15)	
H24	0.328476	0.936589	0.650609	0.068*	
C25	0.4393 (4)	0.8609 (3)	0.6845 (4)	0.0506 (14)	
H25	0.461760	0.872032	0.748095	0.061*	
C26	0.6969 (3)	0.6606 (4)	0.7304 (3)	0.0471 (13)	
H26	0.721891	0.669551	0.794480	0.057*	
C27	0.7377 (4)	0.6055 (5)	0.6938 (5)	0.0650 (17)	
H27	0.792063	0.577059	0.732452	0.078*	
C28	0.7018 (4)	0.5891 (5)	0.6000 (4)	0.0635 (17)	
H28	0.731967	0.549990	0.575834	0.076*	
C29	0.6250 (4)	0.6284 (4)	0.5438 (4)	0.0481 (13)	
H29	0.600529	0.615429	0.480499	0.058*	
C30	0.4988 (4)	0.7306 (4)	0.5209 (3)	0.0405 (12)	
C31	0.5696 (3)	0.7629 (3)	0.7055 (3)	0.0364 (11)	
C32	0.4875 (3)	0.8051 (3)	0.6514 (3)	0.0381 (11)	
C33	0.4527 (3)	0.7886 (3)	0.5551 (3)	0.0400 (12)	
C34	0.5802 (3)	0.6883 (3)	0.5776 (3)	0.0349 (11)	
C35	0.6165 (3)	0.7057 (3)	0.6735 (3)	0.0369 (11)	
C36	0.4597 (4)	0.7112 (3)	0.4208 (3)	0.0413 (12)	
C37	0.3956 (3)	0.6454 (3)	0.3713 (3)	0.0370 (11)	
C38	0.3817 (3)	0.6569 (4)	0.2828 (3)	0.0429 (12)	
C39	0.3211 (4)	0.6178 (4)	0.1946 (3)	0.0478 (13)	
H39A	0.316485	0.552762	0.201431	0.072*	
H39B	0.344798	0.629473	0.149346	0.072*	
H39C	0.261828	0.645184	0.174453	0.072*	
C40	0.3545 (3)	0.5804 (3)	0.4101 (3)	0.0365 (11)	
C41	0.2435 (4)	0.4683 (4)	0.3801 (4)	0.0487 (13)	
H41A	0.226101	0.498471	0.424769	0.058*	
H41B	0.282252	0.416477	0.410479	0.058*	
C42	0.1625 (4)	0.4370 (4)	0.3006 (4)	0.0563 (15)	
H42A	0.180119	0.410741	0.255014	0.084*	
H42B	0.122459	0.488051	0.273756	0.084*	
H42C	0.131623	0.391508	0.320559	0.084*	
O9	0.5612 (9)	0.7525 (10)	0.8442 (5)	0.075 (5)	0.64 (3)
O10	0.6744 (11)	0.8219 (11)	0.8389 (6)	0.076 (4)	0.64 (3)
O9A	0.6187 (17)	0.7124 (9)	0.8578 (8)	0.060 (6)	0.36 (3)
O10A	0.6276 (15)	0.8540 (10)	0.8356 (9)	0.054 (5)	0.36 (3)

### Ethyl 5-methyl-3-(10-nitroanthracen-9-yl)isoxazole-4-carboxylate (I) Atomic displacement parameters (Å^2^)

**Table:**

	*U*^11^	*U*^22^	*U*^33^	*U*^12^	*U*^13^	*U*^23^
O1	0.052 (2)	0.056 (2)	0.0267 (19)	0.0025 (19)	0.0058 (17)	−0.0110 (15)
O2	0.045 (2)	0.058 (2)	0.0280 (19)	0.0043 (17)	0.0083 (16)	−0.0063 (15)
O3	0.037 (2)	0.066 (2)	0.0312 (19)	0.0086 (18)	0.0079 (16)	−0.0030 (17)
N1	0.044 (2)	0.049 (3)	0.029 (2)	0.006 (2)	0.0054 (19)	0.0002 (19)
C1	0.031 (3)	0.041 (3)	0.038 (3)	−0.006 (2)	0.008 (2)	0.003 (2)
C2	0.044 (3)	0.057 (3)	0.055 (4)	−0.015 (3)	0.021 (3)	0.001 (3)
C3	0.050 (3)	0.069 (4)	0.048 (3)	−0.011 (3)	0.023 (3)	0.018 (3)
C4	0.048 (3)	0.060 (3)	0.031 (3)	−0.003 (3)	0.015 (2)	0.006 (2)
C5	0.031 (3)	0.050 (3)	0.026 (2)	−0.009 (2)	0.007 (2)	−0.008 (2)
C6	0.035 (3)	0.053 (3)	0.044 (3)	−0.019 (3)	0.015 (2)	−0.009 (2)
C7	0.044 (3)	0.050 (3)	0.039 (3)	−0.012 (2)	0.020 (2)	0.001 (2)
C8	0.037 (3)	0.046 (3)	0.027 (2)	−0.004 (2)	0.014 (2)	−0.003 (2)
C9	0.029 (2)	0.040 (3)	0.029 (2)	−0.001 (2)	0.0097 (19)	−0.002 (2)
C10	0.032 (2)	0.049 (3)	0.022 (2)	0.000 (2)	0.0062 (19)	−0.001 (2)
C11	0.031 (2)	0.042 (3)	0.032 (2)	−0.002 (2)	0.012 (2)	0.006 (2)
C12	0.028 (2)	0.038 (2)	0.030 (2)	−0.0012 (19)	0.0099 (19)	0.0026 (19)
C13	0.029 (2)	0.033 (2)	0.025 (2)	0.0011 (18)	0.0104 (19)	−0.0022 (18)
C14	0.026 (2)	0.043 (3)	0.029 (2)	−0.001 (2)	0.0112 (19)	−0.005 (2)
C15	0.037 (3)	0.035 (3)	0.024 (2)	−0.007 (2)	0.011 (2)	−0.0030 (19)
C16	0.033 (2)	0.043 (3)	0.023 (2)	−0.006 (2)	0.0047 (19)	−0.004 (2)
C17	0.038 (3)	0.047 (3)	0.026 (2)	−0.005 (2)	0.004 (2)	−0.004 (2)
C18	0.053 (3)	0.073 (4)	0.019 (2)	0.000 (3)	−0.001 (2)	−0.007 (2)
C19	0.041 (3)	0.048 (3)	0.028 (3)	−0.008 (2)	0.011 (2)	−0.003 (2)
C20	0.051 (3)	0.060 (4)	0.045 (3)	0.010 (3)	0.021 (3)	0.002 (3)
C21	0.049 (3)	0.069 (4)	0.050 (4)	0.009 (3)	0.014 (3)	0.009 (3)
O4	0.100 (19)	0.068 (6)	0.035 (2)	0.004 (14)	0.015 (12)	−0.010 (3)
O5	0.031 (6)	0.118 (10)	0.044 (3)	0.013 (5)	0.007 (4)	0.017 (5)
N2	0.037 (10)	0.044 (15)	0.025 (7)	0.008 (10)	0.003 (7)	0.018 (9)
O4A	0.100 (19)	0.068 (6)	0.035 (2)	0.004 (14)	0.015 (12)	−0.010 (3)
O5A	0.031 (6)	0.118 (10)	0.044 (3)	0.013 (5)	0.007 (4)	0.017 (5)
N2A	0.037 (10)	0.044 (15)	0.025 (7)	0.008 (10)	0.003 (7)	0.018 (9)
O6	0.065 (3)	0.058 (2)	0.031 (2)	−0.017 (2)	0.0094 (19)	0.0037 (16)
O7	0.047 (2)	0.052 (2)	0.0246 (17)	0.0098 (17)	0.0108 (15)	0.0032 (14)
O8	0.050 (2)	0.0442 (19)	0.0293 (18)	−0.0018 (17)	0.0132 (16)	−0.0044 (15)
N3	0.063 (3)	0.058 (3)	0.034 (2)	−0.016 (2)	0.008 (2)	−0.003 (2)
N4	0.052 (3)	0.050 (3)	0.032 (2)	−0.015 (2)	0.003 (2)	−0.006 (2)
C22	0.045 (3)	0.056 (3)	0.038 (3)	0.005 (3)	0.005 (2)	0.002 (3)
C23	0.055 (4)	0.053 (3)	0.057 (4)	0.017 (3)	0.010 (3)	0.003 (3)
C24	0.064 (4)	0.045 (3)	0.060 (4)	0.014 (3)	0.024 (3)	−0.002 (3)
C25	0.061 (4)	0.039 (3)	0.045 (3)	−0.005 (3)	0.015 (3)	−0.008 (2)
C26	0.041 (3)	0.059 (3)	0.030 (3)	0.002 (3)	0.004 (2)	0.014 (2)
C27	0.047 (3)	0.085 (5)	0.058 (4)	0.024 (3)	0.016 (3)	0.016 (3)
C28	0.058 (4)	0.085 (5)	0.047 (3)	0.026 (3)	0.021 (3)	0.008 (3)
C29	0.044 (3)	0.067 (4)	0.032 (3)	0.007 (3)	0.014 (2)	0.007 (3)
C30	0.045 (3)	0.041 (3)	0.029 (3)	−0.004 (2)	0.010 (2)	0.000 (2)
C31	0.042 (3)	0.037 (2)	0.023 (2)	−0.008 (2)	0.007 (2)	−0.0019 (19)
C32	0.042 (3)	0.033 (2)	0.034 (3)	−0.005 (2)	0.012 (2)	−0.001 (2)
C33	0.041 (3)	0.038 (3)	0.030 (3)	−0.003 (2)	0.004 (2)	−0.003 (2)
C34	0.035 (3)	0.037 (2)	0.027 (2)	−0.006 (2)	0.007 (2)	0.0038 (19)
C35	0.035 (3)	0.040 (3)	0.028 (2)	−0.009 (2)	0.005 (2)	0.005 (2)
C36	0.048 (3)	0.041 (3)	0.025 (2)	0.004 (2)	0.006 (2)	0.006 (2)
C37	0.039 (3)	0.039 (3)	0.023 (2)	0.005 (2)	0.003 (2)	−0.0026 (19)
C38	0.043 (3)	0.043 (3)	0.032 (3)	−0.002 (2)	0.006 (2)	0.002 (2)
C39	0.052 (3)	0.056 (3)	0.028 (3)	−0.007 (3)	0.009 (2)	−0.002 (2)
C40	0.038 (3)	0.035 (3)	0.034 (3)	0.010 (2)	0.012 (2)	−0.002 (2)
C41	0.051 (3)	0.057 (3)	0.042 (3)	−0.004 (3)	0.024 (3)	−0.001 (2)
C42	0.050 (3)	0.066 (4)	0.054 (4)	−0.002 (3)	0.023 (3)	−0.010 (3)
O9	0.093 (9)	0.100 (9)	0.038 (4)	−0.031 (8)	0.032 (5)	−0.006 (4)
O10	0.065 (7)	0.104 (8)	0.044 (4)	−0.036 (7)	0.008 (5)	−0.009 (5)
O9A	0.100 (16)	0.045 (7)	0.032 (6)	0.009 (7)	0.023 (7)	0.007 (5)
O10A	0.048 (10)	0.047 (8)	0.048 (7)	−0.023 (7)	0.003 (7)	−0.014 (5)

### Ethyl 5-methyl-3-(10-nitroanthracen-9-yl)isoxazole-4-carboxylate (I) Geometric parameters (Å, º)

**Table:**

O1—N1	1.428 (5)	O6—N3	1.418 (6)
O1—C17	1.338 (7)	O6—C38	1.349 (7)
O2—C19	1.199 (6)	O7—C40	1.219 (6)
O3—C19	1.324 (6)	O8—C40	1.319 (6)
O3—C20	1.455 (7)	O8—C41	1.470 (6)
N1—C15	1.318 (6)	N3—C36	1.301 (7)
C1—H1	0.9500	N4—C31	1.474 (6)
C1—C2	1.365 (7)	N4—O9	1.237 (9)
C1—C12	1.423 (7)	N4—O10	1.203 (11)
C2—H2	0.9500	N4—O9A	1.281 (13)
C2—C3	1.401 (8)	N4—O10A	1.206 (14)
C3—H3	0.9500	C22—H22	0.9500
C3—C4	1.366 (8)	C22—C23	1.352 (8)
C4—H4	0.9500	C22—C33	1.420 (8)
C4—C11	1.416 (7)	C23—H23	0.9500
C5—H5	0.86 (6)	C23—C24	1.406 (9)
C5—C6	1.343 (7)	C24—H24	0.9500
C5—C14	1.416 (7)	C24—C25	1.366 (9)
C6—H6	0.85 (6)	C25—H25	0.9500
C6—C7	1.415 (7)	C25—C32	1.403 (8)
C7—H7	0.9500	C26—H26	0.9500
C7—C8	1.367 (7)	C26—C27	1.346 (9)
C8—H8	0.9500	C26—C35	1.425 (7)
C8—C13	1.415 (6)	C27—H27	0.9500
C9—C12	1.408 (7)	C27—C28	1.399 (9)
C9—C13	1.399 (6)	C28—H28	0.9500
C9—C15	1.493 (6)	C28—C29	1.343 (8)
C10—C11	1.380 (7)	C29—H29	0.9500
C10—C14	1.407 (7)	C29—C34	1.406 (7)
C10—N2	1.43 (5)	C30—C33	1.408 (8)
C10—N2A	1.55 (6)	C30—C34	1.416 (7)
C11—C12	1.433 (6)	C30—C36	1.498 (7)
C13—C14	1.436 (6)	C31—C32	1.413 (7)
C15—C16	1.413 (7)	C31—C35	1.388 (7)
C16—C17	1.373 (7)	C32—C33	1.438 (7)
C16—C19	1.485 (7)	C34—C35	1.433 (7)
C17—C18	1.485 (7)	C36—C37	1.415 (7)
C18—H18A	0.9800	C37—C38	1.362 (7)
C18—H18B	0.9800	C37—C40	1.465 (7)
C18—H18C	0.9800	C38—C39	1.474 (7)
C20—H20A	0.9900	C39—H39A	0.9800
C20—H20B	0.9900	C39—H39B	0.9800
C20—C21	1.500 (8)	C39—H39C	0.9800
C21—H21A	0.9800	C41—H41A	0.9900
C21—H21B	0.9800	C41—H41B	0.9900
C21—H21C	0.9800	C41—C42	1.488 (8)
O4—N2	1.22 (6)	C42—H42A	0.9800
O5—N2	1.29 (4)	C42—H42B	0.9800
O4A—N2A	1.27 (9)	C42—H42C	0.9800
O5A—N2A	1.11 (4)		
			
C17—O1—N1	109.5 (3)	O5A—N2A—O4A	131 (6)
C19—O3—C20	114.9 (4)	C38—O6—N3	109.0 (4)
C15—N1—O1	104.3 (4)	C40—O8—C41	116.3 (4)
C2—C1—H1	119.9	C36—N3—O6	105.7 (4)
C2—C1—C12	120.2 (5)	O9—N4—C31	116.7 (5)
C12—C1—H1	119.9	O10—N4—C31	118.0 (6)
C1—C2—H2	119.5	O10—N4—O9	125.2 (7)
C1—C2—C3	120.9 (5)	O9A—N4—C31	118.6 (6)
C3—C2—H2	119.5	O10A—N4—C31	121.6 (8)
C2—C3—H3	119.7	O10A—N4—O9A	119.7 (9)
C4—C3—C2	120.6 (5)	C23—C22—H22	119.6
C4—C3—H3	119.7	C23—C22—C33	120.9 (5)
C3—C4—H4	119.7	C33—C22—H22	119.6
C3—C4—C11	120.7 (5)	C22—C23—H23	119.5
C11—C4—H4	119.7	C22—C23—C24	121.0 (5)
C6—C5—H5	115 (4)	C24—C23—H23	119.5
C6—C5—C14	120.7 (5)	C23—C24—H24	120.0
C14—C5—H5	124 (4)	C25—C24—C23	120.0 (6)
C5—C6—H6	116 (4)	C25—C24—H24	120.0
C5—C6—C7	121.2 (5)	C24—C25—H25	119.4
C7—C6—H6	122 (4)	C24—C25—C32	121.1 (5)
C6—C7—H7	120.2	C32—C25—H25	119.4
C8—C7—C6	119.6 (5)	C27—C26—H26	119.9
C8—C7—H7	120.2	C27—C26—C35	120.2 (5)
C7—C8—H8	119.3	C35—C26—H26	119.9
C7—C8—C13	121.4 (4)	C26—C27—H27	119.3
C13—C8—H8	119.3	C26—C27—C28	121.5 (5)
C12—C9—C15	118.5 (4)	C28—C27—H27	119.3
C13—C9—C12	122.2 (4)	C27—C28—H28	119.8
C13—C9—C15	119.3 (4)	C29—C28—C27	120.4 (6)
C11—C10—C14	125.3 (4)	C29—C28—H28	119.8
C11—C10—N2	121.3 (18)	C28—C29—H29	119.6
C11—C10—N2A	115 (2)	C28—C29—C34	120.9 (5)
C14—C10—N2	113.2 (17)	C34—C29—H29	119.6
C14—C10—N2A	120 (2)	C33—C30—C34	122.7 (4)
C4—C11—C12	118.7 (4)	C33—C30—C36	119.0 (5)
C10—C11—C4	124.3 (4)	C34—C30—C36	118.3 (5)
C10—C11—C12	117.0 (4)	C32—C31—N4	116.4 (4)
C1—C12—C11	118.9 (4)	C35—C31—N4	118.1 (4)
C9—C12—C1	121.6 (4)	C35—C31—C32	125.5 (4)
C9—C12—C11	119.5 (4)	C25—C32—C31	125.2 (5)
C8—C13—C14	117.8 (4)	C25—C32—C33	118.9 (5)
C9—C13—C8	123.2 (4)	C31—C32—C33	115.9 (5)
C9—C13—C14	119.0 (4)	C22—C33—C32	118.1 (5)
C5—C14—C13	119.3 (4)	C30—C33—C22	122.2 (5)
C10—C14—C5	123.7 (4)	C30—C33—C32	119.7 (5)
C10—C14—C13	117.0 (4)	C29—C34—C30	122.7 (4)
N1—C15—C9	119.8 (4)	C29—C34—C35	119.1 (4)
N1—C15—C16	112.6 (4)	C30—C34—C35	118.2 (5)
C16—C15—C9	127.6 (4)	C26—C35—C34	117.8 (5)
C15—C16—C19	126.2 (4)	C31—C35—C26	124.1 (4)
C17—C16—C15	104.2 (4)	C31—C35—C34	118.0 (4)
C17—C16—C19	129.4 (4)	N3—C36—C30	119.9 (5)
O1—C17—C16	109.5 (4)	N3—C36—C37	111.5 (4)
O1—C17—C18	116.7 (4)	C37—C36—C30	128.6 (5)
C16—C17—C18	133.8 (5)	C36—C37—C40	125.7 (4)
C17—C18—H18A	109.5	C38—C37—C36	105.3 (5)
C17—C18—H18B	109.5	C38—C37—C40	129.0 (5)
C17—C18—H18C	109.5	O6—C38—C37	108.6 (4)
H18A—C18—H18B	109.5	O6—C38—C39	115.7 (4)
H18A—C18—H18C	109.5	C37—C38—C39	135.6 (5)
H18B—C18—H18C	109.5	C38—C39—H39A	109.5
O2—C19—O3	124.6 (5)	C38—C39—H39B	109.5
O2—C19—C16	123.0 (5)	C38—C39—H39C	109.5
O3—C19—C16	112.3 (4)	H39A—C39—H39B	109.5
O3—C20—H20A	110.3	H39A—C39—H39C	109.5
O3—C20—H20B	110.3	H39B—C39—H39C	109.5
O3—C20—C21	107.3 (5)	O7—C40—O8	124.2 (5)
H20A—C20—H20B	108.5	O7—C40—C37	123.0 (5)
C21—C20—H20A	110.3	O8—C40—C37	112.7 (4)
C21—C20—H20B	110.3	O8—C41—H41A	110.0
C20—C21—H21A	109.5	O8—C41—H41B	110.0
C20—C21—H21B	109.5	O8—C41—C42	108.4 (5)
C20—C21—H21C	109.5	H41A—C41—H41B	108.4
H21A—C21—H21B	109.5	C42—C41—H41A	110.0
H21A—C21—H21C	109.5	C42—C41—H41B	110.0
H21B—C21—H21C	109.5	C41—C42—H42A	109.5
O4—N2—C10	123 (3)	C41—C42—H42B	109.5
O4—N2—O5	122 (4)	C41—C42—H42C	109.5
O5—N2—C10	116 (3)	H42A—C42—H42B	109.5
O4A—N2A—C10	108 (3)	H42A—C42—H42C	109.5
O5A—N2A—C10	121 (5)	H42B—C42—H42C	109.5
			
O1—N1—C15—C9	−179.2 (4)	N2A—C10—C14—C5	9 (3)
O1—N1—C15—C16	0.1 (5)	N2A—C10—C14—C13	−172 (2)
N1—O1—C17—C16	−0.1 (6)	O6—N3—C36—C30	−179.1 (5)
N1—O1—C17—C18	−179.4 (5)	O6—N3—C36—C37	1.3 (6)
N1—C15—C16—C17	−0.1 (6)	N3—O6—C38—C37	−0.5 (6)
N1—C15—C16—C19	−176.0 (5)	N3—O6—C38—C39	176.6 (5)
C1—C2—C3—C4	−0.7 (9)	N3—C36—C37—C38	−1.6 (6)
C2—C1—C12—C9	179.1 (5)	N3—C36—C37—C40	178.6 (5)
C2—C1—C12—C11	0.6 (7)	N4—C31—C32—C25	0.1 (7)
C2—C3—C4—C11	0.0 (9)	N4—C31—C32—C33	179.7 (4)
C3—C4—C11—C10	−176.7 (5)	N4—C31—C35—C26	1.3 (7)
C3—C4—C11—C12	1.0 (8)	N4—C31—C35—C34	179.0 (4)
C4—C11—C12—C1	−1.2 (7)	C22—C23—C24—C25	0.4 (10)
C4—C11—C12—C9	−179.8 (5)	C23—C22—C33—C30	177.8 (6)
C5—C6—C7—C8	−0.6 (8)	C23—C22—C33—C32	−1.1 (8)
C6—C5—C14—C10	178.2 (5)	C23—C24—C25—C32	−0.6 (9)
C6—C5—C14—C13	−1.0 (7)	C24—C25—C32—C31	179.5 (5)
C6—C7—C8—C13	−0.2 (8)	C24—C25—C32—C33	−0.1 (8)
C7—C8—C13—C9	−176.8 (5)	C25—C32—C33—C22	0.9 (7)
C7—C8—C13—C14	0.3 (7)	C25—C32—C33—C30	−178.0 (5)
C8—C13—C14—C5	0.2 (6)	C26—C27—C28—C29	−0.1 (11)
C8—C13—C14—C10	−179.0 (4)	C27—C26—C35—C31	179.3 (5)
C9—C13—C14—C5	177.5 (4)	C27—C26—C35—C34	1.6 (8)
C9—C13—C14—C10	−1.7 (6)	C27—C28—C29—C34	1.6 (10)
C9—C15—C16—C17	179.1 (5)	C28—C29—C34—C30	−179.5 (5)
C9—C15—C16—C19	3.2 (8)	C28—C29—C34—C35	−1.5 (8)
C10—C11—C12—C1	176.6 (5)	C29—C34—C35—C26	−0.1 (7)
C10—C11—C12—C9	−2.0 (7)	C29—C34—C35—C31	−177.9 (4)
C11—C10—C14—C5	−178.2 (5)	C30—C34—C35—C26	178.0 (4)
C11—C10—C14—C13	1.0 (7)	C30—C34—C35—C31	0.1 (6)
C11—C10—N2—O4	77 (4)	C30—C36—C37—C38	178.9 (5)
C11—C10—N2—O5	−101 (3)	C30—C36—C37—C40	−0.9 (9)
C11—C10—N2A—O4A	115 (4)	C31—C32—C33—C22	−178.7 (5)
C11—C10—N2A—O5A	−64 (6)	C31—C32—C33—C30	2.3 (7)
C12—C1—C2—C3	0.4 (9)	C32—C31—C35—C26	−176.7 (5)
C12—C9—C13—C8	177.7 (4)	C32—C31—C35—C34	0.9 (7)
C12—C9—C13—C14	0.7 (7)	C33—C22—C23—C24	0.5 (10)
C12—C9—C15—N1	91.6 (6)	C33—C30—C34—C29	178.1 (5)
C12—C9—C15—C16	−87.6 (6)	C33—C30—C34—C35	0.1 (7)
C13—C9—C12—C1	−177.3 (5)	C33—C30—C36—N3	95.1 (7)
C13—C9—C12—C11	1.3 (7)	C33—C30—C36—C37	−85.4 (7)
C13—C9—C15—N1	−88.6 (6)	C34—C30—C33—C22	179.7 (5)
C13—C9—C15—C16	92.3 (6)	C34—C30—C33—C32	−1.4 (8)
C14—C5—C6—C7	1.2 (8)	C34—C30—C36—N3	−85.9 (6)
C14—C10—C11—C4	178.5 (5)	C34—C30—C36—C37	93.6 (7)
C14—C10—C11—C12	0.8 (7)	C35—C26—C27—C28	−1.5 (10)
C14—C10—N2—O4	−97 (4)	C35—C31—C32—C25	178.2 (5)
C14—C10—N2—O5	85 (3)	C35—C31—C32—C33	−2.2 (7)
C14—C10—N2A—O4A	−71 (5)	C36—C30—C33—C22	−1.4 (8)
C14—C10—N2A—O5A	110 (5)	C36—C30—C33—C32	177.5 (4)
C15—C9—C12—C1	2.6 (7)	C36—C30—C34—C29	−0.8 (7)
C15—C9—C12—C11	−178.9 (4)	C36—C30—C34—C35	−178.8 (4)
C15—C9—C13—C8	−2.1 (7)	C36—C37—C38—O6	1.2 (6)
C15—C9—C13—C14	−179.2 (4)	C36—C37—C38—C39	−175.0 (6)
C15—C16—C17—O1	0.1 (5)	C36—C37—C40—O7	−3.9 (8)
C15—C16—C17—C18	179.3 (6)	C36—C37—C40—O8	175.1 (5)
C15—C16—C19—O2	−5.4 (8)	C38—O6—N3—C36	−0.5 (6)
C15—C16—C19—O3	173.5 (4)	C38—C37—C40—O7	176.3 (5)
C17—O1—N1—C15	0.0 (5)	C38—C37—C40—O8	−4.6 (7)
C17—C16—C19—O2	179.8 (5)	C40—O8—C41—C42	166.0 (4)
C17—C16—C19—O3	−1.2 (7)	C40—C37—C38—O6	−179.0 (5)
C19—O3—C20—C21	−174.3 (5)	C40—C37—C38—C39	4.7 (10)
C19—C16—C17—O1	175.8 (5)	C41—O8—C40—O7	2.6 (7)
C19—C16—C17—C18	−5.1 (10)	C41—O8—C40—C37	−176.5 (4)
C20—O3—C19—O2	0.5 (7)	O9—N4—C31—C32	63.2 (11)
C20—O3—C19—C16	−178.4 (4)	O9—N4—C31—C35	−115.0 (11)
N2—C10—C11—C4	4.9 (18)	O10—N4—C31—C32	−113.3 (12)
N2—C10—C11—C12	−172.8 (17)	O10—N4—C31—C35	68.5 (13)
N2—C10—C14—C5	−4.1 (18)	O9A—N4—C31—C32	120.0 (14)
N2—C10—C14—C13	175.1 (17)	O9A—N4—C31—C35	−58.2 (15)
N2A—C10—C11—C4	−8 (3)	O10A—N4—C31—C32	−63.1 (16)
N2A—C10—C11—C12	174 (2)	O10A—N4—C31—C35	118.7 (16)

### Ethyl 5-methyl-3-(10-nitroanthracen-9-yl)isoxazole-4-carboxylate (I) Hydrogen-bond geometry (Å, º)

**Table:**

*D*—H···*A*	*D*—H	H···*A*	*D*···*A*	*D*—H···*A*
C1—H1···O5^i^	0.95	2.46	3.366 (12)	159
C3—H3···O7^ii^	0.95	2.44	3.339 (6)	158
C7—H7···O4^iii^	0.95	2.40	3.24 (4)	147
C7—H7···O4*A*^iii^	0.95	2.46	3.34 (6)	154

Symmetry codes: (i) *x*+1/2, −*y*+1/2, *z*+1/2; (ii) *x*, −*y*+1, *z*−1/2; (iii) *x*, −*y*, *z*+1/2.

### Ethyl 3-(9-hydroxy-10-oxo-9,10-dihydroanthracen-9-yl)-5-methylisoxazole-4-carboxylate (V) Crystal data

**Table:**

C_21_H_17_NO_5_	*F*(000) = 760
*M**_r_* = 363.36	*D*_x_ = 1.445 Mg m^−^^3^
Monoclinic, *P*2_1_/*c*	Mo *K*α radiation, λ = 0.71073 Å
*a* = 8.2862 (4) Å	Cell parameters from 9893 reflections
*b* = 23.5895 (11) Å	θ = 2.5–28.3°
*c* = 8.6219 (4) Å	µ = 0.10 mm^−^^1^
β = 97.728 (2)°	*T* = 100 K
*V* = 1669.99 (14) Å^3^	Prism, yellow
*Z* = 4	0.28 × 0.20 × 0.19 mm

### Ethyl 3-(9-hydroxy-10-oxo-9,10-dihydroanthracen-9-yl)-5-methylisoxazole-4-carboxylate (V) Data collection

**Table:**

Bruker SMART Breeze CCD diffractometer	3252 reflections with *I* > 2σ(*I*)
Radiation source: 2 kW sealed X-ray tube	*R*_int_ = 0.051
φ and ω scans	θ_max_ = 28.3°, θ_min_ = 1.7°
Absorption correction: numerical (SADABS; Krause *et al.*, 2015)	*h* = −10→11
*T*_min_ = 0.945, *T*_max_ = 1.000	*k* = −31→31
44252 measured reflections	*l* = −11→11
4112 independent reflections	

### Ethyl 3-(9-hydroxy-10-oxo-9,10-dihydroanthracen-9-yl)-5-methylisoxazole-4-carboxylate (V) Refinement

**Table:**

Refinement on *F*^2^	Primary atom site location: structure-invariant direct methods
Least-squares matrix: full	Hydrogen site location: mixed
*R*[*F*^2^ > 2σ(*F*^2^)] = 0.051	H atoms treated by a mixture of independent and constrained refinement
*wR*(*F*^2^) = 0.114	*w* = 1/[σ^2^(*F*_o_^2^) + (0.0241*P*)^2^ + 1.7289*P*] where *P* = (*F*_o_^2^ + 2*F*_c_^2^)/3
*S* = 1.13	(Δ/σ)_max_ < 0.001
4112 reflections	Δρ_max_ = 0.37 e Å^−^^3^
250 parameters	Δρ_min_ = −0.21 e Å^−^^3^
0 restraints	

### Ethyl 3-(9-hydroxy-10-oxo-9,10-dihydroanthracen-9-yl)-5-methylisoxazole-4-carboxylate (V) Special details

**Table:**

Geometry. All esds (except the esd in the dihedral angle between two l.s. planes) are estimated using the full covariance matrix. The cell esds are taken into account individually in the estimation of esds in distances, angles and torsion angles; correlations between esds in cell parameters are only used when they are defined by crystal symmetry. An approximate (isotropic) treatment of cell esds is used for estimating esds involving l.s. planes.

### Ethyl 3-(9-hydroxy-10-oxo-9,10-dihydroanthracen-9-yl)-5-methylisoxazole-4-carboxylate (V) Fractional atomic coordinates and isotropic or equivalent isotropic displacement parameters (Å^2^)

**Table:**

	*x*	*y*	*z*	*U*_iso_*/*U*_eq_	
O1	0.65863 (16)	0.55415 (6)	0.82793 (16)	0.0252 (3)	
O2	0.18140 (16)	0.55228 (6)	1.16490 (15)	0.0203 (3)	
O3	0.44673 (16)	0.68686 (5)	0.90343 (16)	0.0217 (3)	
O4	0.37570 (18)	0.77723 (6)	0.93929 (18)	0.0306 (3)	
O5	0.03664 (16)	0.70944 (5)	1.19895 (16)	0.0226 (3)	
N1	0.08101 (19)	0.65146 (6)	1.19429 (19)	0.0212 (3)	
C1	0.0870 (2)	0.57205 (7)	0.8333 (2)	0.0203 (4)	
H1	−0.001664	0.578626	0.890399	0.024*	
C2	0.0569 (2)	0.55858 (8)	0.6759 (2)	0.0228 (4)	
H2A	−0.052182	0.555788	0.625751	0.027*	
C3	0.1853 (2)	0.54909 (8)	0.5906 (2)	0.0240 (4)	
H3	0.164699	0.541177	0.481704	0.029*	
C4	0.3430 (2)	0.55131 (7)	0.6659 (2)	0.0218 (4)	
H4	0.431105	0.543796	0.608819	0.026*	
C5	0.7367 (2)	0.57793 (8)	1.1470 (2)	0.0220 (4)	
H5	0.823547	0.568479	1.090262	0.026*	
C6	0.7694 (2)	0.59175 (8)	1.3033 (2)	0.0247 (4)	
H6	0.878586	0.592110	1.353877	0.030*	
C7	0.6426 (2)	0.60514 (8)	1.3866 (2)	0.0243 (4)	
H7	0.665219	0.614443	1.494505	0.029*	
C8	0.4833 (2)	0.60507 (8)	1.3136 (2)	0.0205 (4)	
H8	0.397213	0.614388	1.371534	0.025*	
C9	0.2716 (2)	0.59122 (7)	1.0817 (2)	0.0165 (3)	
C10	0.5456 (2)	0.56479 (7)	0.9026 (2)	0.0189 (4)	
C11	0.5762 (2)	0.57770 (7)	1.0712 (2)	0.0183 (4)	
C12	0.4490 (2)	0.59136 (7)	1.1556 (2)	0.0172 (4)	
C13	0.2460 (2)	0.57606 (7)	0.9088 (2)	0.0172 (4)	
C14	0.3747 (2)	0.56448 (7)	0.8250 (2)	0.0182 (4)	
C15	0.1976 (2)	0.64929 (7)	1.1067 (2)	0.0170 (4)	
C16	0.2349 (2)	0.70473 (7)	1.0516 (2)	0.0181 (4)	
C17	0.1296 (2)	0.73971 (8)	1.1146 (2)	0.0199 (4)	
C18	0.0996 (2)	0.80170 (8)	1.1084 (2)	0.0253 (4)	
H18A	0.008645	0.810745	1.165995	0.038*	
H18B	0.072691	0.813653	0.999147	0.038*	
H18C	0.197593	0.821674	1.156078	0.038*	
C19	0.3576 (2)	0.72714 (8)	0.9602 (2)	0.0194 (4)	
C20	0.5726 (2)	0.70681 (9)	0.8136 (2)	0.0263 (4)	
H20A	0.534468	0.741549	0.755484	0.032*	
H20B	0.593554	0.677531	0.736546	0.032*	
C21	0.7273 (3)	0.71933 (11)	0.9198 (3)	0.0365 (5)	
H21A	0.812088	0.730603	0.856913	0.055*	
H21B	0.762503	0.685366	0.980518	0.055*	
H21C	0.708331	0.750213	0.991184	0.055*	
H2	0.232 (3)	0.5184 (12)	1.162 (3)	0.043 (7)*	

### Ethyl 3-(9-hydroxy-10-oxo-9,10-dihydroanthracen-9-yl)-5-methylisoxazole-4-carboxylate (V) Atomic displacement parameters (Å^2^)

**Table:**

	*U*^11^	*U*^22^	*U*^33^	*U*^12^	*U*^13^	*U*^23^
O1	0.0224 (7)	0.0278 (7)	0.0271 (7)	0.0023 (6)	0.0097 (6)	0.0023 (6)
O2	0.0205 (7)	0.0183 (6)	0.0235 (7)	−0.0023 (5)	0.0081 (5)	0.0017 (5)
O3	0.0199 (7)	0.0209 (6)	0.0256 (7)	−0.0025 (5)	0.0070 (5)	0.0004 (5)
O4	0.0339 (8)	0.0194 (7)	0.0397 (9)	−0.0005 (6)	0.0090 (7)	0.0073 (6)
O5	0.0204 (7)	0.0214 (7)	0.0264 (7)	0.0033 (5)	0.0049 (5)	−0.0018 (5)
N1	0.0187 (8)	0.0192 (8)	0.0262 (8)	0.0018 (6)	0.0047 (6)	−0.0021 (6)
C1	0.0212 (9)	0.0180 (8)	0.0222 (9)	−0.0024 (7)	0.0043 (7)	0.0000 (7)
C2	0.0231 (10)	0.0196 (9)	0.0242 (10)	−0.0046 (7)	−0.0021 (8)	−0.0008 (7)
C3	0.0342 (11)	0.0183 (9)	0.0190 (9)	−0.0041 (8)	0.0017 (8)	−0.0016 (7)
C4	0.0285 (10)	0.0162 (9)	0.0216 (9)	−0.0018 (7)	0.0073 (8)	−0.0011 (7)
C5	0.0169 (9)	0.0218 (9)	0.0279 (10)	0.0000 (7)	0.0047 (7)	0.0079 (7)
C6	0.0172 (9)	0.0256 (10)	0.0300 (11)	−0.0034 (7)	−0.0021 (8)	0.0088 (8)
C7	0.0262 (10)	0.0244 (9)	0.0212 (9)	−0.0020 (8)	−0.0009 (8)	0.0029 (7)
C8	0.0206 (9)	0.0199 (9)	0.0215 (9)	0.0005 (7)	0.0045 (7)	0.0012 (7)
C9	0.0156 (8)	0.0162 (8)	0.0185 (9)	−0.0022 (6)	0.0050 (7)	0.0007 (6)
C10	0.0200 (9)	0.0147 (8)	0.0229 (9)	0.0002 (7)	0.0057 (7)	0.0030 (7)
C11	0.0178 (9)	0.0158 (8)	0.0214 (9)	−0.0011 (7)	0.0034 (7)	0.0037 (7)
C12	0.0168 (9)	0.0135 (8)	0.0210 (9)	−0.0002 (6)	0.0020 (7)	0.0027 (6)
C13	0.0200 (9)	0.0130 (8)	0.0190 (9)	−0.0020 (6)	0.0040 (7)	0.0002 (6)
C14	0.0213 (9)	0.0135 (8)	0.0202 (9)	−0.0011 (7)	0.0047 (7)	0.0008 (6)
C15	0.0143 (8)	0.0196 (8)	0.0168 (8)	−0.0013 (7)	0.0004 (7)	−0.0002 (7)
C16	0.0161 (9)	0.0186 (8)	0.0187 (9)	−0.0005 (7)	−0.0012 (7)	−0.0004 (7)
C17	0.0182 (9)	0.0216 (9)	0.0184 (9)	0.0000 (7)	−0.0028 (7)	−0.0008 (7)
C18	0.0282 (10)	0.0208 (9)	0.0259 (10)	0.0047 (8)	0.0002 (8)	−0.0016 (8)
C19	0.0187 (9)	0.0205 (9)	0.0178 (9)	−0.0010 (7)	−0.0025 (7)	0.0014 (7)
C20	0.0250 (10)	0.0303 (10)	0.0253 (10)	−0.0049 (8)	0.0094 (8)	0.0022 (8)
C21	0.0219 (11)	0.0466 (13)	0.0410 (13)	−0.0056 (9)	0.0040 (9)	0.0122 (10)

### Ethyl 3-(9-hydroxy-10-oxo-9,10-dihydroanthracen-9-yl)-5-methylisoxazole-4-carboxylate (V) Geometric parameters (Å, º)

**Table:**

O1—C10	1.231 (2)	C7—C8	1.383 (3)
O2—C9	1.436 (2)	C8—H8	0.9500
O2—H2	0.91 (3)	C8—C12	1.392 (3)
O3—C19	1.336 (2)	C9—C12	1.521 (2)
O3—C20	1.458 (2)	C9—C13	1.520 (2)
O4—C19	1.208 (2)	C9—C15	1.528 (2)
O5—N1	1.418 (2)	C10—C11	1.474 (3)
O5—C17	1.336 (2)	C10—C14	1.483 (3)
N1—C15	1.304 (2)	C11—C12	1.397 (3)
C1—H1	0.9500	C13—C14	1.393 (3)
C1—C2	1.384 (3)	C15—C16	1.439 (2)
C1—C13	1.392 (3)	C16—C17	1.365 (3)
C2—H2A	0.9500	C16—C19	1.466 (3)
C2—C3	1.391 (3)	C17—C18	1.483 (3)
C3—H3	0.9500	C18—H18A	0.9800
C3—C4	1.380 (3)	C18—H18B	0.9800
C4—H4	0.9500	C18—H18C	0.9800
C4—C14	1.397 (3)	C20—H20A	0.9900
C5—H5	0.9500	C20—H20B	0.9900
C5—C6	1.377 (3)	C20—C21	1.501 (3)
C5—C11	1.401 (3)	C21—H21A	0.9800
C6—H6	0.9500	C21—H21B	0.9800
C6—C7	1.387 (3)	C21—H21C	0.9800
C7—H7	0.9500		
			
C9—O2—H2	106.1 (16)	C8—C12—C9	118.00 (16)
C19—O3—C20	115.78 (15)	C8—C12—C11	119.62 (17)
C17—O5—N1	109.24 (14)	C11—C12—C9	122.37 (16)
C15—N1—O5	105.61 (14)	C1—C13—C9	118.24 (16)
C2—C1—H1	119.7	C1—C13—C14	119.11 (17)
C2—C1—C13	120.57 (18)	C14—C13—C9	122.62 (16)
C13—C1—H1	119.7	C4—C14—C10	119.05 (17)
C1—C2—H2A	119.8	C13—C14—C4	119.83 (17)
C1—C2—C3	120.38 (18)	C13—C14—C10	121.11 (16)
C3—C2—H2A	119.8	N1—C15—C9	117.35 (15)
C2—C3—H3	120.4	N1—C15—C16	111.38 (16)
C4—C3—C2	119.28 (18)	C16—C15—C9	131.27 (16)
C4—C3—H3	120.4	C15—C16—C19	134.48 (17)
C3—C4—H4	119.6	C17—C16—C15	103.91 (16)
C3—C4—C14	120.74 (18)	C17—C16—C19	121.48 (16)
C14—C4—H4	119.6	O5—C17—C16	109.86 (16)
C6—C5—H5	119.8	O5—C17—C18	116.12 (17)
C6—C5—C11	120.49 (18)	C16—C17—C18	134.02 (18)
C11—C5—H5	119.8	C17—C18—H18A	109.5
C5—C6—H6	120.1	C17—C18—H18B	109.5
C5—C6—C7	119.80 (18)	C17—C18—H18C	109.5
C7—C6—H6	120.1	H18A—C18—H18B	109.5
C6—C7—H7	119.8	H18A—C18—H18C	109.5
C8—C7—C6	120.49 (18)	H18B—C18—H18C	109.5
C8—C7—H7	119.8	O3—C19—C16	113.44 (15)
C7—C8—H8	119.9	O4—C19—O3	123.74 (18)
C7—C8—C12	120.15 (18)	O4—C19—C16	122.82 (18)
C12—C8—H8	119.9	O3—C20—H20A	109.5
O2—C9—C12	109.31 (14)	O3—C20—H20B	109.5
O2—C9—C13	109.03 (14)	O3—C20—C21	110.68 (17)
O2—C9—C15	104.91 (14)	H20A—C20—H20B	108.1
C12—C9—C15	108.81 (14)	C21—C20—H20A	109.5
C13—C9—C12	114.27 (15)	C21—C20—H20B	109.5
C13—C9—C15	110.09 (14)	C20—C21—H21A	109.5
O1—C10—C11	121.06 (17)	C20—C21—H21B	109.5
O1—C10—C14	120.73 (17)	C20—C21—H21C	109.5
C11—C10—C14	118.21 (16)	H21A—C21—H21B	109.5
C5—C11—C10	119.19 (17)	H21A—C21—H21C	109.5
C12—C11—C5	119.44 (17)	H21B—C21—H21C	109.5
C12—C11—C10	121.35 (16)		
			
O1—C10—C11—C5	0.1 (3)	C9—C15—C16—C17	−179.04 (17)
O1—C10—C11—C12	178.18 (16)	C9—C15—C16—C19	−3.3 (3)
O1—C10—C14—C4	0.7 (3)	C10—C11—C12—C8	−177.95 (16)
O1—C10—C14—C13	179.53 (16)	C10—C11—C12—C9	2.8 (3)
O2—C9—C12—C8	−57.8 (2)	C11—C5—C6—C7	0.5 (3)
O2—C9—C12—C11	121.43 (17)	C11—C10—C14—C4	−178.84 (16)
O2—C9—C13—C1	54.2 (2)	C11—C10—C14—C13	0.0 (2)
O2—C9—C13—C14	−123.89 (17)	C12—C9—C13—C1	176.78 (15)
O2—C9—C15—N1	1.7 (2)	C12—C9—C13—C14	−1.3 (2)
O2—C9—C15—C16	−179.40 (17)	C12—C9—C15—N1	−115.21 (17)
O5—N1—C15—C9	179.43 (14)	C12—C9—C15—C16	63.7 (2)
O5—N1—C15—C16	0.30 (19)	C13—C1—C2—C3	−0.3 (3)
N1—O5—C17—C16	0.41 (19)	C13—C9—C12—C8	179.71 (15)
N1—O5—C17—C18	−179.49 (15)	C13—C9—C12—C11	−1.0 (2)
N1—C15—C16—C17	−0.1 (2)	C13—C9—C15—N1	118.84 (17)
N1—C15—C16—C19	175.66 (19)	C13—C9—C15—C16	−62.2 (2)
C1—C2—C3—C4	2.3 (3)	C14—C10—C11—C5	179.66 (16)
C1—C13—C14—C4	2.6 (3)	C14—C10—C11—C12	−2.3 (2)
C1—C13—C14—C10	−176.28 (16)	C15—C9—C12—C8	56.2 (2)
C2—C1—C13—C9	179.77 (16)	C15—C9—C12—C11	−124.52 (17)
C2—C1—C13—C14	−2.1 (3)	C15—C9—C13—C1	−60.4 (2)
C2—C3—C4—C14	−1.8 (3)	C15—C9—C13—C14	121.53 (17)
C3—C4—C14—C10	178.25 (17)	C15—C16—C17—O5	−0.22 (19)
C3—C4—C14—C13	−0.6 (3)	C15—C16—C17—C18	179.7 (2)
C5—C6—C7—C8	−0.4 (3)	C15—C16—C19—O3	6.9 (3)
C5—C11—C12—C8	0.1 (3)	C15—C16—C19—O4	−172.96 (19)
C5—C11—C12—C9	−179.15 (16)	C17—O5—N1—C15	−0.44 (19)
C6—C5—C11—C10	177.76 (17)	C17—C16—C19—O3	−178.00 (16)
C6—C5—C11—C12	−0.3 (3)	C17—C16—C19—O4	2.2 (3)
C6—C7—C8—C12	0.2 (3)	C19—O3—C20—C21	86.7 (2)
C7—C8—C12—C9	179.26 (16)	C19—C16—C17—O5	−176.64 (15)
C7—C8—C12—C11	0.0 (3)	C19—C16—C17—C18	3.2 (3)
C9—C13—C14—C4	−179.41 (16)	C20—O3—C19—O4	0.8 (3)
C9—C13—C14—C10	1.7 (3)	C20—O3—C19—C16	−179.03 (15)

### Ethyl 3-(9-hydroxy-10-oxo-9,10-dihydroanthracen-9-yl)-5-methylisoxazole-4-carboxylate (V) Hydrogen-bond geometry (Å, º)

**Table:**

*D*—H···*A*	*D*—H	H···*A*	*D*···*A*	*D*—H···*A*
O2—H2···O1^i^	0.91 (3)	1.93 (3)	2.8359 (19)	176 (2)

Symmetry code: (i) −*x*+1, −*y*+1, −*z*+2.
